# Effects of omega-3 polyunsaturated fatty acid supplementation in patients with chronic chagasic cardiomyopathy: study protocol for a randomized controlled trial

**DOI:** 10.1186/1745-6215-14-379

**Published:** 2013-11-11

**Authors:** Paula S Silva, Gilberto Marcelo Sperandio da Silva, Andréa P de Souza, Claudia SA Cardoso, Cristiane A Fonseca, Patricia D Brito, Roberto M Saraiva, Pedro EA Brasil, Roberta O Pinheiro, Alejandro M Hasslocher-Moreno, Sérgio S Xavier, Andréa S Sousa

**Affiliations:** 1 Serviço de Nutrição, Instituto de Pesquisa Clínica Evandro Chagas, Fundação Oswaldo Cruz, Av. Brasil 4365, Manguinhos, Rio de Janeiro, Brasil; 2 Laboratório de Pesquisa Clínica em Doença de Chagas, Instituto de Pesquisa Clínica Evandro Chagas, Fundação Oswaldo Cruz, Av. Brasil 4365, Manguinhos, Rio de Janeiro, Brasil; 3 Laboratório de Inovações em Terapias, Ensino e Bioprodutos, Instituto Oswaldo Cruz, Fundação Oswaldo Cruz, Av. Brasil 4365, Manguinhos, Rio de Janeiro, Brasil; 4 Laboratório de Hanseníase, Instituto Oswaldo Cruz, Fundação Oswaldo Cruz, Av. Brasil 4365, Manguinhos, Rio de Janeiro, Brasil

**Keywords:** Chagas disease, *Trypanosoma cruzi*, Chronic chagasic cardiomyopathy, Omega-3, Cytokines, Lipid profile, Nutritional assessment

## Abstract

**Background:**

Chronic chagasic cardiomyopathy is an inflammatory disease that occurs in approximately 30% of patients infected by the protozoan *Trypanosoma cruzi*, and it has a profile of high morbidity and mortality. The worst prognosis and the progression of this cardiomyopathy are associated with an exacerbated immune response and the production of proinflammatory cytokines, which also occur in other cardiomyopathies. Some nutrients, including omega-3 polyunsaturated fatty acids (PUFAs), promote the inhibition and/or stimulation of cytokine production. The objective of this trial is to study the effects of omega-3 PUFA supplementation on the inflammatory response and lipid profile in patients with chronic chagasic cardiomyopathy.

**Methods/Design:**

This is a parallel, randomized, placebo-controlled, double-blind clinical trial with 40 patients that will be conducted at a reference unit for Chagas disease patients, where the patients will be selected. The study will include patients with chronic chagasic cardiomyopathy who are 18 years of age or older. The exclusion criteria are (a) ongoing diarrheal disease, (b) inflammatory bowel disease, (c) diabetes or other endocrine disease, (d) use of fibrates, niacin, or statins, (e) use of anti-inflammatory drugs, (f) pregnant and lactating women, (g) use of vitamin, mineral, or omega-3 supplementation during the previous 30 days, (h) hospital admission during the study, and (i) other associated cardiomyopathies. The intervention will be treatment with omega-3 PUFAs at a dose of 3 g/day for 8 weeks, compared to placebo (corn oil). The primary endpoints will be the concentrations of inflammatory markers (interleukin (IL)-1, IL-2, IL-4, IL-6, IL-10, tumor necrosis factor (TNF)α, interferon (IFN)γ, and transforming growth factor (TGF)β). Secondary endpoints will be the fasting glucose, lipid, and anthropometric profiles. For statistical analysis, we plan to run either a t test or Wilcoxon test (numerical variables) and Pearson’s χ^2^ or Fisher’s exact test (categorical data), as appropriate.

**Discussion:**

Evidence suggests that the anti-inflammatory action of omega-3 PUFAs may have beneficial effects on chronic chagasic cardiomyopathy, as shown for other cardiomyopathies, due to improved control of the inflammatory response. At the end of the study, we predict that patients will have lower inflammatory markers and an improved metabolic and anthropometric profile.

**Trial registration:**

Current Controlled Trials NCT01863576

## Background

Chagas disease is endemic in Latin American countries, where approximately 8 to 10 million people are infected with *Trypanosoma cruzi*[1]. In Brazil, the number of infected people is approximately 1.9 million, or 1% of the Brazilian population [2].

The etiologic diagnosis of Chagas disease is performed by detection of the parasite by parasitological methods (direct or indirect) and confirmed by two different serological techniques indirect immunofluorescence and enzyme-linked immunosorbent assay (ELISA) [3].

Chagas disease has two distinct clinical phases: acute and chronic [3]. The chronic phase has three forms: indeterminate, cardiac involvement, and/or digestive involvement [3-5]. In most cases, acute disease is not recognized at onset, and the individual remains asymptomatic with no evidence of damage to any organ but with reactive serology for Chagas disease and low parasitemia during the chronic phase [4]. After two or more decades, 20% to 30% of patients progress to the cardiac stage of the disease, when the majority of the deaths and severe complications related to the disease occur [6-8]. Therefore, chronic chagasic cardiomyopathy (CCC) is an important clinical form of Chagas disease due to its impact on morbidity and its high mortality, worsening of the quality of life, and limitation of the ability to work. It is characterized by a myocarditis with multifocal mononuclear inflammatory infiltrates, varying degrees of fibrosis, constant low-grade tissue parasitism, and low or undetectable parasitemia [9] and it occurs earlier and more severely in males [8,10]. Patients in the chronic cardiac phase may manifest heart failure, ventricular and atrial arrhythmias, atrioventricular blocks, thromboembolism, stroke, and sudden death [2,7,11], which together carry a high economic and mortality burden.

Once settled, CCC is progressive and tends worsen due to overlap with inflammation, cell destruction, and fibrosis. Some hypotheses have been proposed to explain the nature of the cardiac inflammatory reaction, mainly persistent heart immune response and reaction to the parasite [12]. Cytokines are important mediators in the maintenance of the inflammatory process, and they can stimulate or inhibit the immune response [13]. Among the several cytokines studied in *T. cruzi* infection, interferon γ (IFNγ) has been associated with host resistance to infection in both experimental models and humans [14,15]. Transforming growth factor β (TGFβ) production is increased in *T. cruzi* infection, and this increase is most likely related to the fibrotic process observed in CCC [16]. These results suggest that cytokines play essential roles in controlling CCC morbidity [17-19].

Some nutrients are associated with the inhibition and/or stimulation of cytokine production, including polyunsaturated fatty acids (PUFAs). Omega-3 PUFAs (linolenic acid, eicosapentaenoic acid, and docosahexaenoic acid) are found in vegetables (soy, canola, linseed) and cold-water fish (mackerel, sardines, salmon, herring). PUFAs are precursors to the biosynthesis of several important metabolites, especially eicosanoids (prostaglandins, leukotrienes, thromboxanes, lipoxins, and others), synthesized from arachidonic acid [20]. Omega-6 PUFAs (such as linoleic acid: 18:2n-6) and omega-3 PUFAs (such as linolenic acid: 18:3n-3) are fundamental to the body and have important roles in cell metabolism, including influencing membrane fluidity, promoting the release of cytokines, and acting as adhesion molecules on macrophages. Thus, changes in their synthesis and metabolism may be associated with endothelial and hemodynamic changes that contribute to the increase in cardiovascular morbidity and mortality [21].

The fatty acid composition of inflammatory cells can be modified by increasing the intake of marine *n*-3 fatty acids, which leads to a higher content of eicosapentaenoic acid (EPA) and docosahexaenoic acid (DHA). A number of cell culture studies have reported that both EPA and DHA decrease the activation of the transcription factor nuclear factor κB (NFκB) in response to inflammatory stimuli, such as lipopolysaccharide and inflammatory cytokines [22]. Omega-3 PUFAs reduce arachidonic acid content in cell membranes, resulting in the synthesis of eicosanoids that have weaker inflammatory properties than the eicosanoids derived from omega-6 PUFAs [23].

Omega-3 supplementation is safe and well tolerated in infectious disease as demonstrated in HIV-infected patients treated with antiretroviral therapy [24,25], and on host resistance to infection from *Plasmodium berghei* or *Plasmodium falciparum*, the causative agents of malaria [26], which like Chagas disease is a parasitic infection.

Several studies involving supplementation of the diet with marine *n*-3 PUFAs in healthy human volunteers have demonstrated decreased production of tumor necrosis factor α (TNFα), interleukin 1β (IL-1β), IL-2, and IL-6 [22,23,27]. Omega-3 PUFA supplementation for 8 weeks has been associated with an increase of peripheral mononuclear cells with high concentrations of EPA and DHA, which have an immunomodulating effect [28]. In healthy adults, fish oil supplementation at 5 mL/day for 8 weeks reduced the production of IL-6 but did not change the production of IL-10, TNFα, or IFNγ [29]. Long-chain omega-3 PUFA (EPA and DHA) plasma levels are inversely associated with the risk of sudden death [30] and may reduce the risk of ischemic heart disease [31]. EPA and DHA are also involved in the reduction of plasma very-low-density lipoprotein cholesterol (VLDL-c) and triglycerides [32].

A study conducted by the Italian group GISSI-HF at 326 cardiology sites and 31 internal medicine sites selected 6,975 patients with heart failure diagnosis to receive 1 daily capsule containing 1 g of omega-3 PUFAs (n = 3,494) or placebo (n = 3,481) [33]. The total mortality of the supplemented group was 27%, and that of the placebo group was 29% (adjusted HR 0.91; 95.5% confidence interval (CI) 0.833 to 0.998; *P* = 0.041). Hospital admissions due to cardiovascular events were 57% in the omega-3 PUFA group versus 59% in the placebo group (adjusted HR 0.92; 99% CI 0.849 to 0.999; *P* = 0.009). The advantages of *n*-3 PUFAs, which were reduced fatal events and hospital admissions from cardiovascular causes, suggest that they have an effect on the mechanisms leading to progression of heart failure [33].

Supplementation with 1 g/day of omega-3 PUFAs in capsules can decrease cardiovascular events by 10% (death, myocardial infarction, stroke) in coronary arterial disease patients [34]. Some studies that assessed the effect of omega-3 supplementation on the immune response used EPA and/or DHA at 1 g to 9 g per day, similar to the ingestion levels of Greenland Eskimos: 6 to 14 g/day, corresponding to 2.7% to 6.3% of their total calorie intake. The highest doses can reduce arachidonic acid in peripheral mononuclear cells [35].

Studies conducted in heart failure patients have not shown adverse events after omega-3 supplementation [33,36]. In contrast, studies in pregnant women and cancer patients have reported gastrointestinal changes (diarrhea, flatulence, gastroesophageal reflux, nausea), although no differences between the treatment and placebo groups were noted [37,38].

Few studies have evaluated the nutritional status of Chagas patients. Three studies have evaluated patients with the digestive form of the disease. In two of these studies [39,40], there was a high prevalence of malnutrition (>60%). The other study evaluated ten patients diagnosed as having megaesophagus, and only one had malnutrition. The others were eutrophic or overweight [41]. A study performed in São Paulo, Brazil, assessed the nutritional state of 66 Chagas patients and found a high prevalence of overweight and obesity (94%) and large waist circumference (55%). The indeterminate form of the disease was the most prevalent and constituted 71% of cases [42]. There are no reports in the literature on the nutritional assessment of CCC patients.

Supplementation with omega-3 PUFAs may lead to increased bodyweight in patients with heart cachexia, as reported by a study that assessed 14 patients with severe heart failure supplemented with 8 g/day omega-3 PUFAs or placebo [43].

Although several studies have shown the beneficial effects of polyunsaturated fatty acids on inflammatory processes, dyslipidemia, and cardiovascular diseases, there are no reports about food intake and PUFA supplementation in CCC patients. Thus, the objective of this study is to assess the effects of omega-3 PUFA supplementation on the lipid profile and the proinflammatory and anti-inflammatory cytokine profiles in CCC patients.

## Methods/Design

### Study design

This is a parallel, double-blind, placebo-controlled clinical trial with balanced randomization (1:1).

### Settings

The proposed clinical trial will be held at a single site, the Evandro Chagas Clinical Research Institute (IPEC), which is one of the technical scientific units of the Oswaldo Cruz Foundation (Fiocruz). IPEC is a 93-year-old institution that is fully dedicated to clinical research on infectious diseases, including Chagas disease. IPEC is a major reference center for Chagas disease research, care, and training.

### Participants (inclusion and exclusion criteria)

Patients will be recruited from the cardiology ambulatory service at IPEC/Fiocruz, Rio de Janeiro, Brazil, during the period determined for screening until the minimum estimated sample size is reached. All patients had a previous epidemiologic history of Chagas disease confirmed with at least two Chagas serology tests (indirect immunofluorescence and ELISA), and clinical evaluations, electrocardiographic and echocardiographic data obtained recently. Selected patients will be older than 18 years, males and females with CCC at stage B (no heart failure symptoms but with segmental or global left ventricular systolic dysfunction by echocardiogram), stage C (symptomatic heart failure), or stage D (end-stage heart failure), according to the current Brazilian Chagas disease consensus [3]. The following exclusion criteria will be applied: diarrheal disease; inflammatory bowel syndrome; diagnosis of diabetes or other endocrine pathologies; use of fibrates, niacin, or statins; use of anti-inflammatory drugs; pregnant and lactating women; vitamin mineral or omega-3 supplementation during the previous 30 days; hospital admission during the study; and the presence of cardiomyopathies other than CCC.

### Interventions: omega-3 versus corn oil placebo

**Figure 1:**
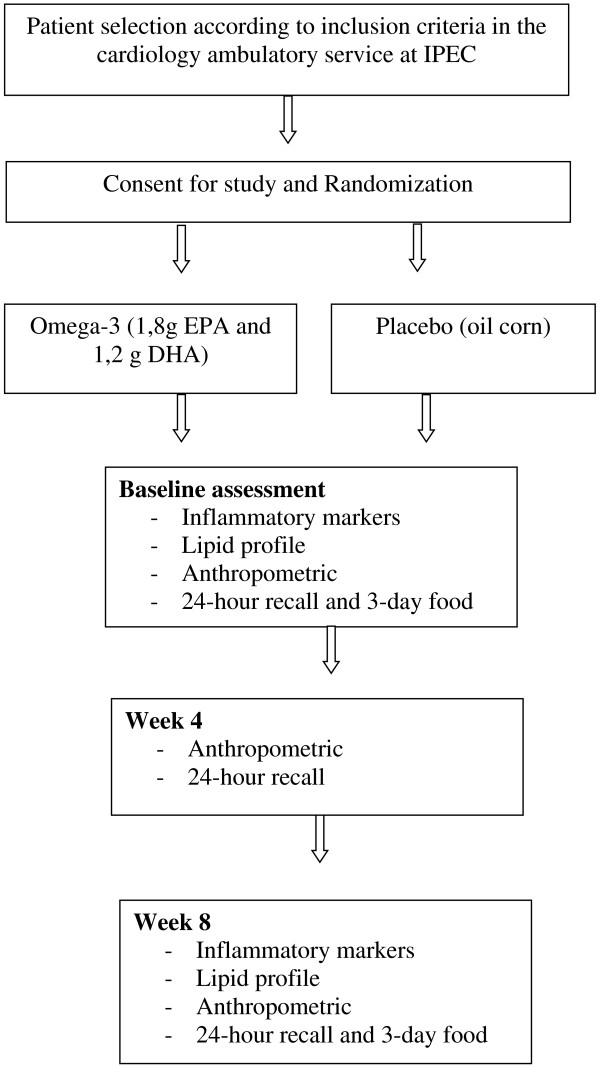
Study flow diagram for omega-3 supplementation in patients with chronic chagasic cardiomyopathy.

Each participant will receive five omega-3 capsules per day (1.8 g EPA and 1.2 g DHA) or placebo for 8 weeks (Figure 1). The omega-3 and corn oil capsules will be provided by Relthy Laboratórios Ltda. (Brazilian Health Surveillance Agency (Agência Nacional de Vigilância Sanitária (ANVISA)) registration: 6.2582.0022.001-1).

### Endpoints

The primary endpoint of this study will be the cytokine profile. IL-1, IL-2, IL-4, IL-6, IL-10, TNFα, IFNγ, and TGFβ will be measured in the serum of patients using specific sandwich enzyme-linked immunosorbent assays. Capture and detection antibodies will be obtained from eBioscience (San Diego, CA, USA). The tests will be conducted according to the manufacturer’s instructions and performed in triplicate.

The first secondary endpoint will be the lipid profile. Total cholesterol, triglycerides, and high-density lipoprotein cholesterol (HDL-c) will be measured with enzymatic colorimetric assays using Siemens reagents on a Siemens Dimension RXL chemistry analyzer (Siemens Healthcare Diagnostics, Tarrytown, NY, USA). Low-density lipoprotein cholesterol (LDL-c) and VLDL-c will be calculated according to the Friedewald equation [44].

Another secondary endpoint of this trial will be anthropometric measures. The anthropometric assessment will consist of body mass index (BMI), waist circumference, tricipital skinfold thickness, and arm circumference [45,46]. BMI will be determined using the standard formula: BMI = weight/height^2^[47].

### Procedures, follow-up, and data collection

After patients are selected by cardiologists, they will be seen by study nutritionists, who will explain the study procedures to the patients and administer the free and informed consent form. The patients who agree to participate in the study will sign the consent form and undergo the initial assessment.

The following data will be collected and evaluated in the study: sociodemographic data (age, sex, ethnicity/race, education, and domicile), clinical data (functional class and vital signs), alcoholism, smoking, prescription drugs, 3-day food record [48], 24-h recall [49], anthropometric assessment (height, weight, BMI, waist circumference, tricipital skinfold thickness, and arm circumference), lipid profile (total cholesterol, triglycerides, HDL-c, LDL-c, and VLDL-c), and cytokines (IL-1, IL-2, IL-4, IL-6, IL-10, TNFα, IFNγ, and TGFβ). Clinical, nutritional, and anthropometric assessments will take place immediately before starting the intervention and after 4 and 8 weeks during the study; lipid profile and cytokines will be evaluated before the intervention and at the end of 8 weeks. Each patient will be followed for 8 weeks.

Compliance with study treatment will be assessed by the 3-day treatment recall and the number of omega-3/placebo capsules returned. At every visit, the patients will be asked about the number of doses of the prescribed treatment that they did not take during the previous 3 days, and the ratio between the number of doses taken and the number prescribed will be calculated. The capsules will be dispensed at the beginning of the study and after 4 weeks, and the returned capsules will be verified at weeks 4 and 8.

The dietary omega-3 PUFA intake will be followed-up during the study (first visit and weeks 4 and 8) by analyzing the 24-h recall and 3-day food record, which are considered indirect methods of analyzing the diet, current dietary standards of a certain population, and their development over time [50].

Patients may be excluded from the study in case of complications during follow-up, such as significant changes in intestinal transit, use of anti-inflammatory drugs, and hospital admissions. Other complications will be assessed by the study team. All endpoints will be collected at time 0 and 8 weeks after omega-3/placebo supplementation starts.

### Safety monitoring

Patients will be followed up by a multidisciplinary team at three time points: study start, week 4, and week 8. Possible complications will be assessed by the study cardiologist and pharmacist. The safety assessment will consist of monitoring adverse events, such as changes in the digestive tract, physical exams, and laboratory tests. Events, onset, and duration will be reported to the cardiologist, who will determine if it is necessary to suspend supplement use. Regular intake of omega-3 PUFAs is not a risk to health. According to the US Food and Drug Administration (FDA), the maximum intake of 3 g/day EPA + DHA is considered safe [51].

### Randomization and concealment

After the screening phase, patients will be randomly assigned to 1 of 2 groups of 20 patients each: intervention group or control group. Assignment to either group will be performed by a computer-generated 1:1 randomization. This assignment will occur within randomly ordered blocks of size 4 or 6. This procedure will be conducted using R project software with package 'blockrand’ [52].

The allocation sequence will be concealed from the researchers, who will enroll and assess participants in sequentially numbered, opaque, sealed, stapled envelopes. To prevent subversion of the allocation sequence, the name and date of birth of the participant will be written on the envelope before the envelope is opened. The information in the envelope will be transferred onto the allocation card inside the envelope. Corresponding envelopes will be opened only after the enrolled participants have completed all baseline assessments and at the time of allocation to the intervention. These procedures will be carried out by a professional without access to the baseline evaluation results. The blister packaging and gel capsules of omega-3 PUFAs and placebo will be identical. They will be identified by the lot number on the package, which will be retained in the pharmacy, and only the blisters will be provided to patients. The pharmacist responsible for distribution and compliance assessment will be the only person who can access this information.

### Blinding

The nutritionists and physician that will perform the initial evaluation before randomization, the monthly evaluations and anthropometric profile, and the final evaluation of the health care questionnaires after the end of the patient follow-up will be blinded to the patients’ assignments.

### Statistical methods

The EpiData [53] and Statistical Package for Social Sciences [54] software will be used for data entry and analysis, respectively. For categorical data, we will use Pearson’s χ^2^ or Fisher’s exact test to verify the association between variables in the two groups (omega-3 and placebo). Statistical analysis will be performed by descriptive analysis, with numerical variables expressed as the mean and standard deviation (SD) or median and interquartile range. The Kolmogorov-Smirnov test will be used to test the normality of the sample distribution. The Student t test will be used in cases with a normal distribution to compare means between the two groups. The Wilcoxon test will be used in cases with a non-normal distribution. *P* values <0.05 will indicate a significant association in all of the statistical tests used.

### Minimum sample size estimation

The minimum estimated sample size is 40 patients according to previous data, considering a prevalence of 60% of inadequate PUFA intake [55]. The following parameters were considered: baseline IFNγ = 3,986 ± 738 pg/mL, IFNγ after *n*-3 PUFA supplementation = 2,922 ± 1,275 pg/mL [56], power = 0.8, and α = 5%. We used the R project software with package 'Epicalc’ version 1.02 [57] to obtain this estimate.

### Ethical issues

The study was approved by the Research Ethics committee of the Evandro Chagas Clinical Research Institute (IPEC/FIOCRUZ), registration number CAAE-0037.0.009.000-10 [58]. It is registered at ClinicalTrials.gov, number NCT01863576. All study supplies and data collected will be used exclusively in this study, and its results will be made public, whether they are favorable or not.

## Discussion

Omega-3 PUFA intake is associated with beneficial health effects. Omega-3 PUFAs are helpful in the prevention and treatment of pathologies such as inflammatory diseases, hypertension, hypertriglyceridemia, metabolic syndrome, and heart diseases. An Italian study of heart failure patients showed a reduction in mortality and hospital admissions due to cardiovascular events, suggesting beneficial effects of omega-3 PUFAs on the progression of heart disease [33].

Omega-3 administration is effective at treating hypertriglyceridemia. According to clinical trials, supplementation with 2 to 4 g of EPA/DHA per day may reduce triglyceride levels by 25% to 30% [59] by decreasing the liver synthesis of VLDL-c [34]. Omega-3 supplementation for chronic chagasic cardiomyopathy may be similar to other cardiomyopathies and promote reductions in triglyceride and VLDL-c levels.

Omega-3 has beneficial effects against heart cachexia after 18 weeks of supplementation [43]. Women with polycystic ovarian syndrome and BMI between 25 and 40 kg/m^2^, however, exhibited no significant changes in BMI after supplementation for 8 weeks [60]. The period of treatment in this study may not be long enough to observe significant anthropometric changes after omega-3 administration with no other associated instruction or intervention.

The action of omega-3 PUFAs is most likely related to their anti-inflammatory effects, which are not yet completely understood; however, it is known that omega-3 PUFA supplementation reduces arachidonic acid content in cell membranes, resulting in the synthesis of eicosanoids that are less inflammatory than the ones derived from omega-6 PUFAs [23]. Omega-3 PUFAs can also decrease activation of NFκB in response to inflammatory stimuli, such as lipopolysaccharide and inflammatory cytokines [22]. A meta-analysis of the effects of fish oil supplementation on the inflammatory markers of patients with chronic heart failure showed that circulating TNFα, IL-1, and IL-6 (proinflammatory cytokines) decreased after a supplementation period of 3 to 12 months with an EPA and DHA dose of 600 to 5,540 mg/day. These data suggest that supplementation with higher doses of omega-3 PUFAs or longer follow-up times are associated with more substantially decreased cytokines [61], which could benefit CCC patients.

A clinical trial evaluated 3-month omega-3 PUFA supplementation in patients with heart failure. Patients were divided into three groups: placebo, 1 g/day omega-3 PUFAs, and 4 g/day omega-3 PUFAs. Only the 4 g/day group had an improvement in ventricular function with an increase in the ejection fraction and endothelial function and a reduction in IL-6 [36]. We will use 3 g/day omega-3 PUFAs in our study based on recent results suggesting higher efficacy when compared to the usual 1 g dose, in addition to the fact that 3 g/day is considered safe by the FDA [51]. According to a recent American College of Cardiology and American Heart Association (ACC/AHA) Heart Failure Guideline, omega-3 supplementation is reasonable to use as adjunctive therapy in heart failure patients (Class of Recommendation IIa, Level of Evidence B ) [62].

The worse prognosis and progression of CCC are related to increased immune response with the production of proinflammatory cytokines and the inhibition of anti-inflammatory cytokines, which also occur in other cardiomyopathies [63,64]. In Chagas disease, heart tissue is an important target of *T. cruzi* infection, and substantial amounts of proinflammatory cytokines, chemokines, and enzymes, including inducible nitric oxide synthase and metalloproteinases, are produced, resulting in inflammation and cardiac remodeling in response to parasite infection [65]. Higher levels of IFNγ and TNFα are associated with lower levels of IL-4 and IL-10, in both cardiac and more severe forms of Chagas disease, and high levels of IL-10 or moderate levels of IFNγ are associated with the indeterminate form [15,66]. Omega-3 and omega-6 PUFA intake may lead to an increase in IL-10 production, as shown in an experimental study in Wistar rats supplemented with ethyl-eicosapentaenoic acid and ethyl-γ-linolenic acid compared to a group supplemented with palm oil [67]. Thus, omega-3 PUFA supplementation in CCC patients may lead to increased IL-4 and IL-10, reducing the inflammatory effect on heart tissue.

Omega-3 PUFA supplementation, due to its anti-inflammatory effects, is expected to decrease the serum levels of proinflammatory cytokines, which are associated with the proliferation of parasites and the fibrotic process characteristic of Chagas myocarditis. We thus suppose that the benefits to CCC patients with regard to the immunological profile could translate into a less severe progression of cardiomyopathy, with subsequent reduction in morbidity, in addition to secondary gains in the lipid profile.

## Trial status

This study is currently recruiting patients.

## Competing interests

The authors declare that they have no competing interests.

## Authors’ contributions

PSS, APdS and ASS conceived the study. GMSdS, AMH-M, PEAB, RMS, SSX, PSS, APdS and ASS participated in the study design. PSS, CSAC, CAF and PDB will recruit, select, and collect clinical data of the patients. PSS, ROP and GMSdS will randomize patients into the two arms of the protocol, deliver the proposed intervention, and collect data. PSS, PEAB, GMSdS and ASS will perform statistical analysis. APS is the study coordinator and ASS the principal investigator. PSS, GMSdS, PEAB, AMH-M and ASS helped to draft the manuscript. All authors read and approved the final manuscript.
